# The formal EU‐US Meniscus Rehabilitation 2024 Consensus: An ESSKA‐AOSSM‐AASPT initiative. Part II—Prevention, non‐operative treatment and return to sport

**DOI:** 10.1002/ksa.12689

**Published:** 2025-06-12

**Authors:** Robert Prill, C. Benjamin Ma, Stephanie E. Wong, Philippe Beaufils, Juan Carlos Monllau, Elanna K. Arhos, Roland Becker, Francesco Della Villa, J. Brett Goodloe, James J. Irrgang, Jitka Klugarova, Emma L. Klosterman, Aleksandra Królikowska, Aaron J. Krych, Robert F. LaPrade, Robert Manske, Nicky van Melick, Jill K. Monson, Marko Ostojic, Mark V. Paterno, Tomasz Piontek, Simone Perelli, Alexandre Rambaud, James Robinson, Laura C. Schmitt, Eric Hamrin Senorski, Thorkell Snaebjornsson, Adam J. Tagliero, Airelle O. Giordano, Nicolas Pujol

**Affiliations:** ^1^ Department of Orthopedics and Traumatology, Brandenburg Medical School Theodor Fontane University Hospital Brandenburg/Havel Brandenburg an der Havel Germany; ^2^ Brandenburg Medical School Theodor Fontane Faculty of Health Sciences Brandenburg Brandenburg an der Havel Germany; ^3^ Department of Orthopaedic Surgery University of California San Francisco California USA; ^4^ Centre Médical, ESSKA Office Luxembourg; ^5^ Department of Orthopedics and Traumatology Hospital del Mar Barcelona Spain; ^6^ Universitat Autònoma de Barcelona, Institut Català de Traumatologia i Medicina del'Esport (ICATME) ‐ Hospital Universitari Dexeus, Knee and Arthroscopy Unit Barcelona Spain; ^7^ Department of Physical Therapy and Human Movement Sciences Northwestern University Chicago Illinois USA; ^8^ Isokinetic Medical Group, FIFA Medical Centre of Excellence, Education & Research Department Bologna Italy; ^9^ Department of Orthopedic Surgery VCU Health Richmond Virginia USA; ^10^ Department of Physical Therapy University of Pittsburgh School of Health and Rehabilitation Sciences Pittsburgh Pennsylvania USA; ^11^ Cochrane Czech Republic, Czech CEBHC: JBI Centre of Excellence, Czech GRADE Network, Institute of Health Information and Statistics of the Czech Republic Prague Czech Republic; ^12^ Department of Orthopaedic Surgery Michigan Medicine Ann Arbor Michigan USA; ^13^ Physiotherapy Research Laboratory University Centre of Physiotherapy and Rehabilitation, Faculty of Physiotherapy, Wroclaw Medical University Wroclaw Poland; ^14^ Department of Orthopedic Surgery Mayo Clinic Rochester Minnesota USA; ^15^ Twin Cities Orthopedics, Edina and Eagan Minnesota USA; ^16^ Wichita State University, Ascension Via Christi Wichita Kansas USA; ^17^ Sports & Orthopedics Research Center, Anna TopSupport Eindhoven The Netherlands; ^18^ Osteon Orthopedics and Sports Medicine Clinic Mostar Bosnia and Herzegovina; ^19^ Division of Occupational Therapy and Physical Therapy, Division of Sports Medicine Cincinnati Children's Hospital Medical Center Cincinnati Ohio USA; ^20^ Department of Pediatrics University of Cincinnati College of Medicine Cincinnati Ohio USA; ^21^ Department of Rehasport, Sport Medicine Lab, Department of Spine Disorders and Pediatric Orthopedics University of Medical Sciences Poznan Poland; ^22^ Saint‐Michel Campus, Institute of Physiotherapy of Saint‐Etienne Saint‐Etienne France; ^23^ MS Knee Specialists Bristol UK; ^24^ Division of Physical Therapy Ohio State University, School of Health and Rehabilitation Sciences and OSU Sports Medicine Research Institute Columbus Ohio USA; ^25^ Department of Health and Rehabilitation, Institute of Neuroscience and Physiology, Sahlgrenska Academy University of Gothenburg Gothenburg Sweden; ^26^ Medical Faculty, University Hospital of Iceland, Landspitali University of Iceland Reykjavik Iceland; ^27^ Department of Physical Therapy University of Delaware Newark Delaware USA; ^28^ Department of Orthopedic and Trauma Surgery Centre Hospitalier de Versailles Le Chesnay‐Rocquencourt France

**Keywords:** knee, meniscectomy, non‐operative treatment, physical therapy, physiotherapy, repair

## Abstract

**Purpose:**

Part two of this consensus aimed to provide recommendations for the prevention of meniscus injuries, non‐operative treatment of acute tears and degenerative lesions, return to sports and patient‐reported outcome measures.

**Methods:**

This consensus followed the European Society of Knee Surgery, Sports Traumatology and Arthroscopy (ESSKA) formal consensus methodology. For this combined ESSKA—American Orthopedic Society for Sports Medicine (AOSSM)—American Academy of Sports Physical Therapy (AASPT) initiative, 67 experts from 14 countries, including orthopedic surgeons and physiotherapists, were involved. The 26 Steering Group members established guiding questions, screened the existing evidence, and proposed statements, and provided Grades of recommendations. The 41 Rating Group members assessed the statements according to a Likert scale (1–9). Final documents were assessed by an international peer review group for geographical adaptability.

**Results:**

Low to moderate scientific level of evidence was available, so that grades of recommendations were low (three Grade A ratings, four Grade B, three Grade C and 13 Grade D), underlining the relevance of this consensus. One strong and 17 relative agreements with overall median of 8 (8–9) and a mean of 7.92 ± 0.37 were achieved for 23 statements on 18 questions. Prevention of meniscus injuries is possible with general knee injury reduction programmes and through avoidance of certain activities. Non‐operative treatment including physical therapy is the first line approach for degenerative meniscus lesions and may be an option for some acute tears. Return to sports after meniscus tear surgery should be both criterion‐based and time‐based. Patient reported outcomes in combination with performance‐based measures are recommended to evaluate the rehabilitation process.

**Conclusion:**

This international EU–US consensus established recommendations for prevention strategies, describes rehabilitation of non‐operated patients and of patients after partial meniscectomy, meniscus repair and meniscus reconstruction, and establishes return to sport criteria. These updated and structured recommendations may be applied by surgeons and physiotherapists.

**Level of Evidence:**

Level I, consensus.

**Abbreviations**
AASPT: American Academy of Sports Physical Therapy
ACL: anterior cruciate ligament
ADL: activities of daily living
APM: arthroscopic partial meniscectomy
BMI: body mass index
ESSKA: European Society of Knee Surgery, Sports Traumatology, and Arthroscopy
IKDC: International Knee Documentation Committee
KOOS: Knee Injury and Osteoarthritis Outcome Score
MRI: magnetic resonance imaging
NSAID: non‐steroidal anti‐inflammatory drug
OA: osteoarthritis
OMEX RCT: OMEX Randomised Controlled Trial
PBMS: performance‐based measures
PEP: Prevent Injury and Enhance Performance Program
PROMs: patient‐reported outcome measures
RCT: randomized controlled trial
ROM: range of motion
VAS: Visual Analog Scale
WOMAC: Western Ontario and McMaster Universities Arthritis Index

## INTRODUCTION

Meniscectomy is one of the most frequently performed orthopedic surgeries in the world [7]. The long‐term results, even following arthroscopic partial meniscectomy, are heterogenous [8]. The concept of meniscal preservation has progressed over the years with more clear indications: non‐operative management of degenerative meniscus lesions, repair of some traumatic meniscus tears, and meniscus replacement if indicated. There is a wide range of words used to describe pathological meniscus changes, mainly divided into degenerative and acute etiologies. Within the European Society of Knee Surgery, Sports Traumatology and Arthroscopy (ESSKA) European meniscus consensus group, traumatic meniscus injuries are defined as meniscus tears caused by an acute injury mechanism and ‘meniscus lesions’ are degenerative meniscus changes marked by a slow progression [3]. Two ESSKA consensuses are available on the management of acute meniscus tears, and degenerative lesions [3, 16]. Beside the described surgical decision‐making processes, appropriate rehabilitation of meniscus injuries must be emphasised. The fact that two consensuses on surgical treatment exist, the high value of rehabilitation, limited evidence and some controversial topics underline the immense importance of this consensus. The aim of this consensus is to provide recommendations for the usage of rehabilitation (including physical therapy) for patients undergoing either non operative or surgical treatment for degenerative meniscus lesions or acute meniscus tears. In Part I, Rehabilitation after Meniscus Surgery is described and the relevant rehabilitation questions are addressed [18]. Part II covers injury prevention, non‐operative treatment, and return to sport recommendations.

The full‐text of this consensus may be accessed on the ESSKA website: https://esskaeducation.org/esska-consensus-projects.

## MATERIALS AND METHODS

**Figure 1:**
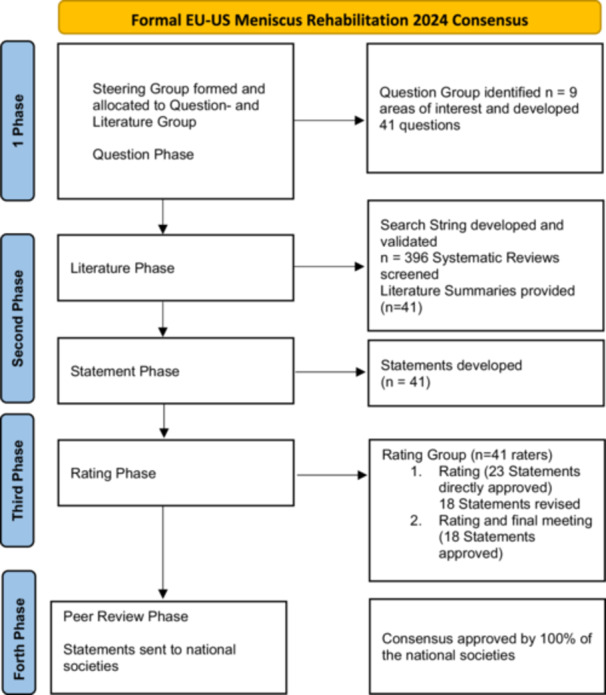
Flowchart.

This consensus followed the ESSKA formal consensus methodology [4, 5]. The first Consensus involving societies from Europe and the United States on a Meniscus topic was established. Between 2022 and 2024, ESSKA, the American Orthopedic Society for Sports Medicine (AOSSM) and the American Academy of Sports Physical Therapy (AASPT) equally contributed to this project. A steering group of four chairs, additionally 26 Steering group members, half Physical Therapists and half Surgeons, from Europe and the US, and project advisors designed questions, performed the literature search, and proposed statements. An additional international rating group (*n* = 41) did a two‐round rating of the proposed statements, according to a Likert scale (0–9) and commented on the statements based on the following format for each question: question, proposed statement, proposed grade of recommendation, specific literature summary. This rating phase was followed by a combined meeting involving Steering and Rating groups where disagreements were solved by discussion. The process for the whole consensus (Part I + II) is shown in Figure 1.

Each statement was followed by the agreement based on the median of raters scores and by the Grade of recommendation. Grade A represents a high level of scientific support, Grade B scientific presumption, Grade C low level of scientific support, and Grade D expert opinion [5].

If a statement was rated 0, this indicates that the rating group found it to be inappropriate or unacceptable, and if it was rated 9, there was complete agreement. A proposal was deemed appropriate when the value of the median was ≥7 and the score of each rater was ≥5, indicating relative agreement, and strong agreement when the value of the median was ≥7, with no singular rater score <7. The mean and median were calculated for each question. A revised manuscript draft was prepared and resubmitted to the rating group for a second assessment for any statements where the median agreement was below 7.

Finally, representatives of the ESSKA national societies assessed the whole consensus for applicability in their geographical setting in Europe. Geographical appropriateness was not considered to be relevant for assessment in the USA: Further details are described in Part 1 of the consensus.

## RESULTS

No questions were deleted for inappropriateness. Of the statements relevant to this paper three received a Grade A rating, four a Grade B, three a Grade C, and 13 a Grade D. Thus, low to moderate scientific level of evidence was available in the current literature. Nine questions went to a revision and second rating. The overall median of all ratings in statements presented in this paper was 8 (8–9), with a mean of 7.95 ± 0.31, (7.16–8.39), resulting in one strong and 17 relative agreements. All statements have been approved by the peer review group and, as a result, by all involved national societies.

### Prevention of meniscus injuries

#### Are any promising attempts to reduce TRAUMATIC meniscus tears currently available?

Lower extremity injury risk reduction programmes focusing on neuromuscular control exercises can be used to prevent overall lower extremity injury, however it is not specific to acute meniscal tears. Prevent Injury and Enhance (PEP), FIFA11+, and Knäkontroll are some examples of useful programmes in lower extremity and anterior cruciate ligament (ACL) risk reduction (Grade C).

Relative Agreement: Mean 7.95 ± 1.63, median 8.5 (5–9)

#### What activities including activities of daily living (ADL) and sporting activities may increase the risk of meniscus tears or lesions?

Participation in repetitive cutting, pivoting and landing activities increases risk of meniscal injury in athletes. Work related lifting and carrying (over 10 pounds/4.5 kg), kneeling, deep squatting, and a high volume of climbing has increased risk of meniscus injury (Grade A).

Relative Agreement: Mean 8.38 ± 0.85, median 8.5 (5–9)

### Rehabilitation management of NON‐OPERATED acute meniscus tears

#### Does non‐operative management benefit the treatment of TRAUMATIC meniscus tears?

Research investigating non‐operative interventions for TRAUMATIC meniscus tears is rare. While allocation to conservative treatment for acute meniscus tears might be an option, allocation criteria for conservative versus surgical interventions are not well established. Two studies, focusing on self‐reported patient outcomes suggest that both surgery and exercise therapy are viable treatment options for TRAUMATIC AND NON‐TRAUMATIC meniscus tears (Grade A).

More severe symptoms and larger tears may benefit from surgical intervention. Bucket handle and complete radial meniscus tears along with extended RAMP lesions and meniscal root tears in younger patients may require earlier surgical intervention to optimise outcomes (GRADE D).

Relative Agreement for both statements: Mean 7.85 ± 1.62, median 8 (5–9)

#### Which components and/or personal factors influence rehabilitation effectiveness in TRAUMATIC meniscus tears?

Evidence is lacking regarding which factors affect rehabilitation of traumatic meniscus tears. Effectiveness may be influenced by factors such as lower extremity alignment, body mass index (BMI), medical comorbidities, psychosocial and socioeconomic factors, use of tobacco products, age, occupation, compliance and level of activity. High grade osteoarthritis (OA), the type, location, and magnitude of the meniscus tear may also play a role in the effectiveness of rehabilitation (GRADE D).

Relative Agreement: Mean 8.04 ± 1.08, median 8 (6–9)

#### What rehabilitation interventions are best indicated for management of non‐operative TRAUMATIC meniscus tears?

The efficacy of various interventions to treat impairments following knee ligament injury have been previously studied but are not specific to meniscus injury. Specific interventions implemented to resolve knee effusion, reduce pain, restore quadriceps strength, and regain joint‐specific motor control may be advantageous in treating TRAUMATIC meniscus tears (i.e., cryotherapy, open and closed‐kinetic‐chain exercise, transcutaneous electrical nerve stimulation, neuromuscular electrical stimulation, exercise with blood flow restriction) (GRADE D).

There is no evidence comparing rehabilitation modalities. At 12 months follow up, a single 2024 study (the‐DREAM trial) showed in TRAUMATIC meniscus tears (stable knees) that supervised neuromuscular and strength training along with patient education produced similar self‐reported outcomes to surgery with the same rehabilitation programme GRADE B).

Relative Agreement on both statements: Mean 7.16 ± 1.90, median 8 (5–9)

#### Is there an evidence‐based non‐operative treatment protocol for treating TRAUMATIC meniscus tears?

A 12‐week supervised neuromuscular exercise programme that includes lower extremity strengthening, balance, hip and core strengthening along with patient education has been found to have similar outcomes to those that underwent surgery for TRAUMATIC meniscus tears with the same rehabilitation protocol.

However, there is no current evidence comparing evidence‐based treatment protocols for treating acute meniscus tears non‐operatively. When choosing non‐operative treatment for acute meniscus tear management, one may follow criterion‐based milestones like those followed for post‐operative meniscal repairs (GRADE B).

Relative Agreement: Mean 7.55 ± 1.86, median 8 (5–9)

#### Is outpatient rehabilitation superior to home exercise programmes for non‐operatively managed TRAUMATIC meniscal tears?

There is no current evidence comparing outpatient rehabilitation programmes to home exercise programmes after TRAUMATIC meniscus tears. Supervised rehabilitation for ROM, effusion management, lower extremity and quadriceps strength and function, and neuromuscular control, in addition to a home exercise programme may be recommended (GRADE D).

Relative Agreement: Mean 8.26 ± 1.20, median 9 (5–9)

### Rehabilitation management of non‐operated degenerative meniscal lesions

#### Is non‐operative management as beneficial as arthroscopic partial meniscectomy for treating symptomatic DEGENERATIVE meniscus lesions?

Degenerative meniscus lesions can be treated with comparable results with either non‐operative (including physical therapy) or surgical approach. Therefore, non‐operative treatment including physical therapy should be the first approach. In case of persistent symptoms despite non‐operative treatment, arthroscopic partial meniscectomy might be considered (GRADE A).

Previous surgical meniscus consensus recommends 3–6 months of non‐operative treatment prior to surgical decision making.

Today, prognosis classifications assessing severity of degenerative meniscus lesions and severity of symptoms are missing and needed. This could help to determine the timing of surgery (GRADE D).

Relative Agreement for both statements: Mean 7.87 ± 1.61, median 8 (5–9)

#### What components, and personal factors of a DEGENERATIVE meniscus lesion make rehabilitation most effective?

There is no evidence to support or refute that knee specific factors or personal factors increase or decrease the likelihood of successful outcome with rehabilitation after degenerative meniscus lesion (including degenerative flap or unstable tears). However, high grade of OA, high BMI, and longer duration of symptoms may negatively influence the outcomes (GRADE D).

Strong Agreement: Mean 8.23 ± 0.59, median 8 (7–9)

#### What rehabilitation treatment is best indicated for the management of non‐operated degenerative meniscus lesions?

Manual therapy, joint mobilisation techniques, ROM exercises, progressive knee and hip musculature strength training, and neuromuscular training may be applied. Also, neuromuscular electrical stimulation can be added. Homebased exercise programmes should be added to supervised physical therapy (GRADE B).

When not contraindicated, blood flow restriction training can be used to enhance early strengthening efforts, maximise low intensity exercise or manage symptoms associated with exercise.

Knee bracing can be considered for symptom management and/or an improved perception of joint stability (Grade D).

Relative Agreement for both statements: Mean 7.62 ± 1.12, median 8 (5–9)

#### Is outpatient rehabilitation superior to home‐based exercise programmes for non‐operatively managed DEGENERATIVE meniscal lesions?

Supervised outpatient rehabilitation and home‐based exercise have not been compared. Supervised rehabilitation for ROM, effusion management, muscle strength, knee function and neuromuscular control, in addition to a home exercise programme may be recommended (GRADE D).

Relative Agreement: Mean 8.00 ± 1.03, median 8 (5–9)

### Return to sports after meniscus injury and surgery

#### What are the criteria and the time for return to sports after management of meniscus tears or lesions?

Return to sports management after meniscus tears or lesions should be criterion based, and time based. Healing time frames should also be considered.

A criterion‐based return to sport progression is recommended for patients after conservative or surgical treatment of meniscus tears or lesions.

The main criteria to consider are subjective and objective knee function, physical and psychological factors. Knee function includes range of motion (ROM), joint effusion, quadriceps and hamstring muscle strength and activation. Performance based parameters are coordination and stabilisation‐based tasks. Patient's motivation and readiness to return to sport needs to be evaluated. However, these criteria are not specific for traumatic meniscus tears.

Besides the criterion‐based time frame, the recommended time to return to sport after meniscus surgery varies depending on the surgical procedure, concomitant injuries and the type of sports. Return to sport is recommended 4–12 weeks for partial meniscectomies, and 6–9 months for meniscal repair (GRADE C).

Relative Agreement: Mean 7.68 ± 1.39, median 8 (5–9)

#### Is on‐field rehabilitation suggested for athletes willing to return to sports after management of meniscus tears or lesions?

Sport‐specific rehabilitation on the field is recommended to complete the on‐field rehabilitation phase. The specific requirements of the sport do matter when making progressive return to sport rehabilitation decisions (GRADE D).

Relative Agreement: Mean 8.08 ± 1.25, median 9 (5–9)

#### What patient‐related and knee‐related factors influence return to sports after meniscus treatment?

Patient‐related and knee‐related factors have an impact on return to sport. Type, location, size of the tear or lesion, type of surgery, concomitant injuries and muscle function might be more important factors determining the time to return to sport (GRADE D).

Relative Agreement: Mean 8.13 ± 1.21, median 9 (5–9)

#### Are activity outcomes dependent on the type of tear (e.g., complex, medial, lateral, repaired, resected, etc.?) When is return to sports not recommended after a meniscus injury or surgery?

Activity outcomes can be dependent on the type of tear or lesion, treatment and whether the patient desires to return to sports.

If knee function cannot be restored and clinical milestones cannot be met, return to sports is not recommended after a meniscus injury or surgery (GRADE D).

Relative Agreement: Mean 7.84 ± 1.60, median 8 (5–9)

### Patient‐reported outcome measures (PROMs) and assessments

#### What patient‐reported outcomes should be used to evaluate treatment for meniscal tear or lesion and/or meniscal surgery?

Rehabilitation after meniscal tear or lesion and/or meniscal surgery should be evaluated using patient‐reported outcomes that cover disease specific questionnaires (recommended: Western Ontario and McMaster Universities Arthritis Index [WOMAC]), knee‐related questionnaires (recommended: International Knee Documentation Committee [IKDC] or Knee Injury and Osteoarthritis Outcome Score [KOOS]), activity‐related questionnaires (recommended: Tegner or Marx activity scale), and pain scales (recommended: Visual Analog Scale [VAS]) (GRADE B).

Agreement: Mean 8.39 ± 1.02, median 9 (5–9)

#### Which clinical exams or functional performance‐based measures (PBMS) are useful to objectify the rehabilitation process?

Rehabilitation after meniscal tear or lesion and/or surgery may be evaluated in a criterion‐based approach (initial‐intermediate‐activity‐return to sport) and include assessments of ROM, knee‐joint effusion, objective strength test (isokinetic or handheld dynamometry), and hop tests to objectify the rehabilitation process.

It could functionally be evaluated with strength tests (recommended: quadriceps/knee extension and hamstrings/knee flexion strength) and activity‐related testing (recommended: hop for distance) and interpreted with the limb symmetry index (GRADE D).

Relative Agreement: Mean 7.78 ± 1.20, median 8 (5–9)

#### What persisting signs and/or symptoms during rehabilitation require a referral to a surgeon?

Patients should be referred to an orthopedic surgeon in cases of persistent pain, recurrence of stiffness and/or effusion, persistent functional instability, mechanical symptoms, or unexpected neurological symptoms (GRADE C).

The inability to reach clinical milestones related to knee symptoms indicates a referral to the orthopedic surgeon (GRADE D).

Relative Agreement for both statements: Mean 8.24 ± 1.0, median 9 (6–9)

## DISCUSSION

The most important findings of this collaborative work of three specialist societies in the field of treating acute meniscus tears and degenerative lesions are important pathways and recommendations regarding prevention, non‐operative treatment and return to sport. Surgical decision making for meniscus pathology has been published in two previous consensus projects and rehabilitation after surgery is covered in Part I of this consensus [3, 16, 18].

No specific meniscus injury prevention programmes exist; therefore evidence comes from general injury prevention programmes or those related to ACL injury prevention strategies [1]. Prevention includes the participation in general knee injury reduction programmes like Prevent Injury and Enhance (PEP), FIFA11+, and Knäkontroll [11, 21]. Even if not developed specifically for the meniscus, the preventive effect of those programmes for meniscal tears and lesions is supported by the literature. Also supported by strong evidence is activity modification, such as avoidance of pivoting and cutting sports, and limiting activities that load the meniscus including lifting, deep squatting or kneeling. Knowledge of these programmes and activity guidelines are crucial for the protection of the menisci [2, 22].

Tears are often classified as either traumatic or degenerative based on factors such as the patient's age, injury mechanism, chronicity of the symptoms, tear pattern, and the presence or absence of arthritic changes in the joint [23]. Few data suggests the potential for conservative treatment of acute meniscus tears [10]. This idea mainly relies on one trial and there is limited to no evidence to describe specific factors influencing the success of rehabilitation for traumatic meniscus tears in terms of conservative treatment alone. First of all, traumatic meniscus tears associated with ACL inury and isolated traumatic tears must be distinguished. Regarding ACL injuries, let the meniscus alone in case of small tears (especially on the lateral side) has been advocated for a long time [16, 17]. There is also the idea of self‐healing potential and of potential thresholds for ramp lesion repair [20]. As far as isolated tears are concerned, there is heterogeneity in tear pattern, location, and type of tear, while precisely described during arthroscopic surgery, it cannot be provided that detailed with clinical examination and MRI. Therefore, it is almost unknown which types tears do better with rehabilitation or surgery and how each of the factors influence the outcome. Currently there is no literature comparing different rehab protocols for acute traumatic tears; physical therapy treatment options can only be chosen to address symptoms like resolving knee effusion, pain modulation, restoring ROM and quadriceps strength. Manual therapy and gentle joint mobilisation have long been used and might be helpful to address acute locking or to reduce pain [9, 19]. Unfortunately, limited knowledge about the type of tear with only clinical or MRI‐based classifications make general recommendations difficult. While certain acute tears like root or radial tears benefit from early surgery, exercise and education are of low risk for other tears and likely beneficial interventions. A 12‐week supervised neuromuscular exercise programme that includes lower extremity strengthening, balance, hip and core strengthening along with patient education has been found to have similar outcomes to those who underwent surgery for traumatic meniscus tears with the same rehabilitation protocol [10]. Exercise is the most commonly studied physical therapy intervention for the non‐operative management of meniscus tears. Generally, because of limited evidence there were controversial discussions on that topic with all statements being partially revised during the rating and revision phase. Research to improve the robustness of this literature is needed.

In 2016, ESSKA published a consensus of the surgical management of degenerative meniscus lesions, suggesting an initial non‐operative treatment period of at least 3 months making the understanding of rehabilitation relevant for all degenerative meniscal lesions [3]. Not all degenerative meniscus lesions cause symptoms, but when they are symptomatic the initial treatment should be non‐operative. The evidence supporting non‐operative treatment options is stronger than in traumatic tears and results between surgery and non‐operative treatment are equivalent [13, 24, 25]. Physical therapy is therefore the first line treatment for three to six months. The statement on this topic achieved very high acceptance in the rating group and was approved without any revision. The general time frame of conservative treatment is partially a result of a lack of prognostic classifications regarding the type of degenerative meniscus lesions and severity of symptoms. In a seven‐center RCT with patients also with osteoarthritis (OA) no difference was observed in pain relief assessed after 6 and 12 months, but thirty percent from conservative group needed surgery at a later timepoint [15]. The recently published 10 year follow‐up of the OMEX RCT supports the idea of equal results for exercise therapy versus APM [6]. Two systematic reviews have failed thus far to predict components and personal factors for predicting who benefits most from arthroscopic partial meniscectomy or conservative treatment [12, 24]. As a comment from the Consensus Group, it must be added that rehabilitation is not the only non‐operative treatment option. There are other possible treatments as rest, NSAID, intraarticular or peri‐meniscal injections. However, they are out the scope of this consensus and their usefulness (in conjunction or in place of rehabilitation) will not be discussed.

There were high levels of agreement on criteria for return to sports after meniscus tears or lesions. Return to sports management should be criterion‐based, especially after meniscus repair surgery, and also time‐based such as healing times have to be respected. Consensus was reached on subjective and objective knee function, physical and psychological factors. The current rate of return‐to‐play following isolated meniscus repair ranges between 71.2% and 100% within a time frame of 3.3–10 months [14].

While a wide range of patient reported outcome measures PROMS are available, WOMAC together with IKDC or KOOS, and MARX or Tegner scores, in combination with clinical assessments of ROM, knee joint effusion and objective strength tests like isokinetic or hop tests, are the most relevant to assessing rehabilitation process of meniscus tears or lesions.

There are some limitations of this consensus, which are mainly related to limited available evidence, supporting some of the statements. There is a strong need, for further investigating optimal treatment approaches in patients with acute meniscal tears. As some of them experience severe pain in the acute phase, non‐operative treatment may be difficult to be recommended, and neuromuscular training will not be an option in the first weeks. Therefore, further studies on early‐stage treatment as well as classification systems, which should probably include symptoms, type of tear, OA status, aimed activity level and rehabilitation speed are needed, as it is currently hard to predict who will benefit from non‐operative or surgical treatment. Additionally, specific rehabilitation protocols need to be compared to each other and to surgical options in prospective trials to strengthen either the idea of specific conservative treatment or of surgery for different types of acute meniscal tears.

## CONCLUSION

This international EU‐US formal consensus, developed by three societies, established sufficient recommendations for prevention strategies, describes rehabilitation of non‐operated patients and of patients after partial meniscectomy, meniscus repair and meniscus reconstruction, and establishes return to sport criteria. Including the recommendations from part 1 of this consensus most relevant clinical questions on rehabilitation of meniscus tears and lesions have been answered. These updated and structured recommendations may be applied by surgeons and physiotherapists.

## AUTHOR CONTRIBUTIONS

Robert Prill wrote the first draft of the manuscript. Robert Prill, Ben Ma, Nicolas Pujol and Airelle Giordano chaired the consensus and have been involved in all parts of the work. Stephanie Wong revised the first draft of this manuscript and was a member of the Steering Group. Phillipe Beaufils is the official ESSKA consensus projects advisor, who advised adhering to the official consensus method. Juan Carlos Monllau was the ESSKA Vice‐president and advised in case of conflicts. All other authors were members of the Steering Group and either developed questions and statements or did sufficient literature work. They are listed in alphabetical order. All authors were involved in the validation process of the statements. All authors read, revised and approved the final draft of this paper.

## CONFLICT OF INTEREST STATEMENT

Nicolas Pujol: occasional consultant for education for Smith & Nephew, Stryker, ZimmerBiomet. Philippe Beaufils: ESSKA Consensus Projects advisor. Stephanie Wong: Editor‐in‐Chief of Current Reviews in Musculoskeletal Medicine. Robert Prill: Chair of the ESSKA Rehabilitation Committee, Associate Editor KSSTA, occasional Consultant OPED GmbH. Airelle O. Giordano: Education Chair, AASPT. Robert F. LaPrade: Consultant: Smith and Nephew, Ossur; Royalties; Smith and Nephew, Ossur, Elsevier; Research support: AOSSM, AANA, Ossur, Smith and Nephew, Arthrex; Editorial Boards: AJSM, KSSTA, JEO, Journal of Knee Surgery; JISPT, OTSM; AOSSM: Nominating Committee; ISAKOS: Travelling Fellowship Committee, Programme Committee. Aaron J. Krych: Consulting and Royalties Arthrex, Inc. Editorial Boards: AJSM, KSSTA. AANA Board. James J. Irrgang: Currently serve as President of the Board of Directors of Movement Media Sciences/Journal of Orthopaedic & Sports Physical Therapy. Alexandre Rambaud: Member of ESSKA Rehabilitation Committee; Deputy Editor: European Rehabilitation Journal, Journal de Traumatologie du Sport, Editor‐in‐Chief of Kinésithérapie, la Revue. Laura C. Schmitt: Editorial Board: MSSE; Research Support from Arthritis Foundation and NFL Players Association. Jill K. Monson: Member AASPT Research Committee, Consultant Smith & Nephew. Mark V. Paterno: Co‐Chair AASPT Research Committee, Research Support from AOSSM/AIrCast Research Foundation. Simone Perelli: Paid consultancy for Smith & Nephew. Aleksandra Królikowska: Member of ESSKA Rehabilitation Committee. Nicky van Melick: Member of ESSKA Rehabilitation Committee. Jitka Klugarová: Deputy director of the Czech CEBHC: JBI Centre of Excellence, Czech GRADE Network, member of methodological group withing JBI and GRADE working group. The other authors did not declare any conflict of interest.

## ETHICS STATEMENT

Not needed for systematic review and consensus statements not involving human participants.

## Data Availability

The data that support the findings of this study are available on request from the corresponding author. The data are not publicly available due to privacy or ethical restrictions. The full text of the Consensus is available on the ESSKA Homepage.
